# Perceived barriers to adopting more plant-based diets across sociodemographic groups: findings from a population-based survey in Finland

**DOI:** 10.1017/jns.2026.10101

**Published:** 2026-05-20

**Authors:** Laura Sares-Jäske, Laura Paalanen, Heli Tapanainen, Niina Eerika Kaartinen, Minna Kaljonen, Hanna Konttinen, Satu Männistö

**Affiliations:** 1 Finnish Institute for Health and Welfare, Finland; 2 Finnish Environment Institute, Finland; 3 University of Helsinki, Finland

**Keywords:** Climate change, Dietary change, Perceived barriers, Plant-based diets, Sociodemographic differences

## Abstract

Plant-based diets are favourable for both climate and health. However, not much is known about different barriers to adopting more plant-based diets across sociodemographic groups. This study examined the proportions of the population reporting a shift towards more plant-based diets and identified perceived barriers to these changes across different sociodemographic groups. The study was based on the Healthy Finland Survey 2022–2023, including 5390 participants aged 20–74 years. Participants responded to questions on plant-based diets using pre-defined response options. Results were presented as prevalences and 95% CIs. Women reported that they had changed their diets to more plant-based more often than men did (46% vs. 31%). Urban residents, higher educated participants, and those with higher income were more likely to have adopted more plant-based diets than others. Concerns about nutritional adequacy (women 20%; men 24%) and lacking skills to prepare tasty vegetarian foods (women 30%; men 28%) were common perceived barriers. In men, barriers included a dislike of vegetarian foods (23%) and a lack of interest (28%). In women, 17% cited the preferences of their close ones as a barrier. Barriers also differed between groups. Older participants were more concerned about the nutritional adequacy (29%), while younger participants struggled with cooking skills (35%). One third of men living in rural areas or with basic education reported a lack of interest in the topic. Tailored health promotion and food education strategies are critical to overcoming barriers to adopting plant-based diets in diverse sociodemographic groups.

## Introduction

Plant-based diets offer significant benefits for both climate and health,^(1,2)^ while diets rich in animal-based foods have negative impacts on both.^(2–4)^ The concept of plant-based diets varies from veganism to diets containing also animal-based foods,^(5)^ but generally the common factor is the attempt to somehow restrict the consumption of animal-based products. Despite red meat consumption in Europe has slightly declined, the average consumption remains relatively high.^(6)^ In Finland, for instance, the majority of men exceed recommended weekly consumption of red and processed meat; 79% of men exceeded the previous recommendation of maximum of 500 g of red and processed meat per week,^(7)^ while 93% of men exceed the new recommendation of max 350 g per week.^(8)^

Food production and consumption contribute to climate change, but climate change also affects people, for example, psychologically. The European Social Survey (ESS) showed that in 2020–2022, at least two-thirds of people in European countries expressed some degree of concern about climate change.^(9)^ This concern may motivate actions like decreasing car use or meat consumption. Changing beliefs, attitudes, and habits is a challenging and time-consuming process.^(10)^ To enable dietary changes at population level, external circumstances and supportive systems are needed to facilitate the changes and make them accessible for individuals.^(11)^ A European consumer survey conducted in 2023, including 7500 participants from 10 countries, showed that 51% of them reported to have reduced meat consumption.^(12)^ However, the share of the participants frequently consuming meat remained unchanged between 2021 and 2023.

Various perceived barriers may hinder dietary changes. Dietary habits are often learned in childhood and deeply rooted into cultural norms. A recent systematic review grouped several individual barriers under themes: financial, lack of knowledge, emotional, health, convenience, social, enjoyment of meat, environmental, accessibility, personal ability, and media.^(13)^

A Finnish study identified meat enjoyment, eating routines, health conceptions, and difficulties in preparing vegetarian food as key barriers to following a plant-based diet,^(14)^ while a Danish study identified concerns about protein content, satiety, and taste as barriers and found that individuals with high consumption of animal-based foods disagreed with statements about positive environmental and health effects of plant-based diets.^(15)^ Other studies found unfamiliarity, price, and taste,^(16)^ as well as practical and cooking concerns along with incompatibility with current diet^(17)^ as barriers to pulse consumption. The lack of knowledge about the climate benefits of reducing meat consumption, identifying sustainable foods, and preparing tasty plant-based food are considerable barriers to adopting plant-based diets.^(18–20)^ A qualitative study found uncertainty in British adults about what changes to make, despite willingness to adopt more sustainable diets.^(21)^ In a study including ten European countries, only half of consumers were aware of the health and environmental benefits of plant-based foods, indicating a knowledge gap.^(12)^ This suggests that more comprehensive information on diet–health–environment associations and practical guidance on plant-based diets are needed.

Previous literature has demonstrated a clear sociodemographic gradient in dietary choices, with women, urban residents, and those with higher education or income to be more likely to adopt healthier and more sustainable diets.^(22–24)^ It is likely that also barriers to adopting more plant-based diets differ across population groups. Men, younger adults, rural residents, those living in households with children, with low education, or low income tend to be generally less interested in reducing meat consumption or adopting plant-based diets than others.^(14,20,25)^ However, in Finland, women perceived more barriers to pulse consumption than men.^(16)^ Although previous studies have identified various barriers that hinder the dietary changes in the whole study populations or in a few population groups, as well as various population groups that generally perceive more barriers, a nuanced understanding of how different barriers differ by various population groups and how they may change over time across countries is inadequate. As barriers may differ depending on individuals’ background, actions of various types may be needed. A recent review concluded that tailored approaches, such as price incentives, normative messaging, and increased accessibility of plant-based foods, that also take into account the specific needs and backgrounds of the individuals, could be effective strategies to decrease meat consumption.^(26)^ To be able to target the measures effectively according to different backgrounds, it is essential to understand which barriers hinder the dietary shift in which population groups. More nuanced knowledge of these group-specific differences can be utilised to help different groups to overcome the barriers and to tailor health promotion and food education strategies that are effective in various sociodemographic groups with different motivations, needs, and situations.

The aims of this study were to examine the proportion of the Finnish adult population that have shifted towards more plant-based diets to combat climate change. Furthermore, we identified perceived barriers to adopting more plant-based diets in different sociodemographic groups, including sex, age, urbanisation level, household structure, education, and household income.

## Methods

### Study participants

This study utilised data from the Healthy Finland Survey 2022–2023.^(27,28)^ The sampling for the survey was conducted with a stratified random sample design to enhance representativeness of the results (for further information, see^(27,28)^). The aims of the survey were to produce comprehensive information on health, well-being, and service use. The information was collected through questionnaires and health examinations. The nationally representative sample comprised 60,861 individuals aged 20 and older permanently living in Finland. A total of 46% (*n* = 28,153) of the sample participated in the survey. A representative sub-sample of 16,080 individuals was invited to participate in a sub-module on climate change. This study analysed 5390 participants aged 20–74 years, who responded to this sub-module and had answered to at least one of the questions related to the dietary changes (variables used as outcomes in this study, described in detail later). Participants who had not answered to any of these questions of interest were excluded from the analyses. Further, depending on the variable, 0-761 individuals had missing information in some of the exposure or outcome variables used.

The Healthy Finland Survey was performed in accordance with the Declaration of Helsinki and approved by the ethics committee of Helsinki and Uusimaa hospital district. All participants gave their written informed consent.

### Measures

The questions concerning dietary changes towards more plant-based diets and potential perceived barriers to these changes were selected and formulated by a group of researchers at the Finnish Institute for Health and Welfare based on a literature search and on questions used in the previous studies. Dietary changes towards more plant-based diets were collected with the question ‘What are you doing or planning to do over the following year to combat climate change?’. One of the 11 pre-defined actions was ‘Change my diet to more plant-based’. The response options included ‘I am doing this or have already done it / I plan to do this / I do not yet plan to do this / does not apply to me’. Uncertainty about environmentally friendly foods was asked with the statement ‘I am unsure about what food is environmentally friendly’, and perceived social pressure to increase vegetarian food consumption was asked with the statement ‘People close to me think I should increase my vegetarian food intake’ representing normative influences of the close ones. Response options for these questions were ‘strongly disagree / somewhat disagree / neither agree nor disagree / somewhat agree / completely agree’. For this study, categories ‘somewhat agree’ and ‘completely agree’ were combined and reported together.

Perceived barriers to adopting plant-based diets were asked with the question ‘Do you feel that any of the following prevents you from making your diet more plant-based?’. Participants could select multiple options. Those who already follow a vegetarian/vegan diet were instructed to skip this question. The pre-defined barriers were:

- I am unsure about whether a plant-based diet is nutritious enough
- I do not know how to prepare tasty vegetarian food
- I cannot afford to have a more plant-based diet
- Plant-based products are not easily available
- I do not have time to change my eating habits
- I do not like vegetarian foods
- The preferences of those close to me prevent me from making vegetarian food
- I am not interested in the topic
- Some other reason.

In this study, barriers were reported both separately and as combined.

Sex, age, urbanisation level of residential area, household structure, education, and household income were used to represent sociodemographic and socio-economic status. Information on sex, age, and urbanisation level were obtained from the survey’s sampling frame and originally from the Digital and population data services agency of Finland. As information on sex was based on official registered sex, categories used in this study (women, men) do not necessarily match perceived gender identity of the participants. Age was categorised into four categories (20–39/40–54/55–64/65–74 years). The age group cut-offs were selected roughly to represent younger adulthood (20–39), lower middle-age (40–54), higher middle-age (55–64), and retirement age (65–74), that are usually characterised by different life events. Additionally, age was used as a 10-year category variable when serving as a confounding factor. Information on residential area was based on coordinates of the participants’ residence locations, which had been categorised according to their level of urbanisation as follows: urban areas/areas near urban areas, rural centres/remote rural areas. For the household structure, education, and household income variables, information was collected with questionnaires. Household structure was categorised as follows: households with only one adult living alone/households with at least two adults and no underage children/households with at least one adult and at least one underage child. Education was based on self-reported number of years of full-time studying including primary school. The number of years was categorised into three tertiles separately in men and in women and in 10-year age groups: low (the lowest tertile)/ intermediate (middle tertile)/high (the highest tertile). The income quartile variable was based on questions about total household income during the last year before tax deductions, and on number of adult and underage household members. The original household income question comprised five pre-defined categories: less than 15,000 €/15,001–35,000 €/35,001–55,000 €/55,001–75,000 €/more than 75,000 €. In this study, upper limits of the categories (and in the highest category, lower limit multiplied by two) were divided by weighted sum of household members, given a value of 1.0 to the first adult, value of 0.7 to additional adults, and value of 0.5 to the underage household members using the OECD equivalence scale.^(29,30)^ The result was further categorised into sex-specific quartiles.

### Statistical methods

All analyses were conducted separately for women and for men due to significant sex differences in most of the studied factors and due to sex differences in diets found in previous studies.^(24,31)^ Results were presented as prevalences and 95% CIs of factors related to adopting a more plant-based diet (outcome variables one at a time) in categories of selected sociodemographic and -economic factors (exposure variables one at a time) (Tables 1–2, Supplementary tables 1–5). The analyses were adjusted for age in 10-year categories (except for the analyses where age was used as an exposure variable (Table 2)) by using Surveyreg procedure in SAS. The prevalences were produced with least squares means which present predicted population margins – that is, they estimate the marginal means over a balanced population. Significant differences in prevalences between sociodemographic groups were evaluated by non-overlapping 95% CIs and by pair-wise tests. In pair-wise tests, multiple comparisons were taken into account using the Studentized Maximum Modulus (SMM) adjustment for multiple comparisons with row-wise denominator degrees of freedom. Survey weights (inverse probability weights), based on register data, were used to mitigate bias caused by non-participation and selection probability and to improve the representativeness of the results to the Finnish adult population.^(28,32)^ All analyses were conducted with SAS Enterprise Guide, version 7.15 HF7 (SAS Institute Inc., Cary, NC, USA).

**Table 1.** Characteristics (population-weighted prevalence and 95% CI) of study population (Healthy Finland survey, *n* = 5,390)

	Women		Men	
	*n*	% (95% CI)	*n*	% (95% CI)
*Sociodemographic information*				
Age (years)	3045		2345	
20–39	668	34.2 (31.8–36.6)	437	35.2 (32.2–38.2)
40–54	710	27.1 (25.0–29.1)	495	28.0 (25.3–30.6)
55–64	733	19.1 (17.6–20.7)	634	19.3 (17.5–21.1)
65–74	934	19.6 (18.1–21.0)	779	17.5 (16.0–19.1)
Urbanisation level of residential area	3045		2345	
Urban areas	1739	65.9 (64.0–67.8)	1295	64.9 (62.5–67.3)
Areas near urban areas, rural centres	779	21.2 (19.5–22.8)	599	21.3 (19.3–23.3)
Remote rural areas	527	13.0 (11.7–14.2)	451	13.8 (12.3–15.4)
Household structure	2650		2031	
Living alone	783	30.5 (28.2–32.8)	567	32.6 (29.7–35.5)
Adults only	1235	40.7 (38.4–43.1)	1006	39.3 (36.4–42.1)
At least one adult and one child	632	28.7 (26.5–31.0)	458	28.1 (25.4–30.9)
Education	2988		2301	
Basic	1166	41.5 (39.2–43.8)	945	41.5 (38.7–44.3)
Intermediate	986	29.7 (27.6–31.7)	702	31.3 (28.7–34.0)
High	836	28.9 (26.7–31.0)	654	27.1 (24.6–29.7)
Household income quartiles	2616		2013	
1st (lowest)	520	24.0 (21.7–26.2)	536	30.5 (27.6–33.4)
2nd–3th	1269	44.5 (42.1–46.9)	891	41.1 (38.1–44.1)
4th (highest)	827	31.5 (29.3–33.8)	586	28.4 (25.8–31.0)
*Questions concerning plant-based diets*				
Changing diet to more plant-based	3028		2324	
Already done that	1385	46.3 (44.0–48.6)	691	30.5 (27.8–33.2)
Plans to do that	645	20.2 (18.4–22.1)	425	15.9 (14.0–17.7)
Don’t yet plan to do that	877	28.7 (26.6–30.8)	975	41.1 (38.3–43.9)
Not applicable	121	4.73 (3.62–5.84)	233	12.5 (10.5–14.5)
Unsure about what food is environmentally friendly	617/3004	21.9 (19.9–23.9)	530/2313	23.4 (20.9–25.9)
Perceived social pressure to increase vegetarian food consumption	322/3001	11.0 (9.43–12.6)	522/2312	19.9 (17.8–22.0)
Barriers that prevent one from making one’s diet more plant-based	3045		2345	
Unsure about whether a plant-based diet is nutritious enough	660	20.3 (18.4–22.1)	579	23.7 (21.3–26.1)
Doesn’t know how to prepare tasty vegetarian food	874	29.7 (27.6–31.8)	627	28.0 (25.6–30.5)
Can’t afford to have a more plant-based diet	336	12.9 (11.2–14.6)	225	12.0 (10.0–13.9)
Plant-based food products are not easily available	119	4.66 (3.41–5.92)	77	3.69 (2.56–4.81)
Doesn’t have time to change eating habits	182	6.78 (5.57–7.98)	139	7.28 (5.83–8.74)
Doesn’t like vegetarian foods	244	8.89 (7.55–10.2)	506	23.4 (20.9–25.9)
The preferences of those close to one prevent from making vegetarian food	508	16.9 (15.2–18.6)	65	3.21 (2.30–4.11)
Not interested in the topic	337	11.0 (9.56–12.4)	635	28.3 (25.7–30.9)
Some other reason	455	15.1 (13.4–16.7)	304	12.6 (10.8–14.3)

**Table 2.** Prevalences and 95% CIs of studied factors related to adopting a more plant-based diet across age groups in women and in men (*n* = 5,390)*

	Women									
	Age								General test P-value	Pair-wise comparison sign. diff.^†^
	20–39 (1)		40–54 (2)		55–64 (3)		65–74 (4)			
	n	% (95% CI)	n	% (95% CI)	n	% (95% CI)	n	% (95% CI)		
Changing diet to more plant-based	666		706		728		928			
Already done that	301	46.0 (41.3–50.7)	319	44.5 (40.0–48.9)	342	48.9 (44.5–53.3)	423	47.0 (43.2–50.8)	NS	NS
Plans to do that	125	18.5 (14.7–22.3)	135	19.6 (16.0–23.3)	160	21.2 (17.7–24.6)	225	23.1 (20.0–26.2)	NS	NS
Don’t yet plan to do that	212	30.0 (25.7–34.4)	220	30.4 (26.3–34.4)	198	26.5 (22.7–30.4)	247	26.3 (23.0–29.7)	NS	NS
Not applicable	28	5.46 (3.11–7.81)	32	5.55 (3.18–7.91)	28	3.42 (1.83–5.01)	33	3.61 (2.19–5.03)	NS	NS
Unsure about what food is environmentally friendly	172/664	26.6 (22.2–30.9)	129/708	19.1 (15.5–22.7)	126/727	17.9 (14.6–21.3)	190/905	21.3 (18.2–24.5)	0.015	1>3
Perceived social pressure to increase vegetarian food consumption	57/666	10.5 (7.08–13.9)	69/707	10.1 (7.36–12.8)	78/725	11.5 (8.65–14.4)	118/903	12.7 (10.2–15.2)	NS	NS
Barriers that prevent one from making one’s diet more plant-based ^‡^	668		710		733		934			
Unsure about whether a plant-based diet is nutritious enough	142	20.5 (16.8–24.2)	110	15.3 (12.0–18.6)	144	18.9 (15.6–22.2)	264	28.0 (24.6–31.5)	<0.0001	1,2,3<4
Doesn’t know how to prepare tasty vegetarian food	245	33.8 (29.4–38.2)	231	32.2 (28.1–36.3)	191	26.9 (23.0–30.8)	207	21.9 (18.8–25.0)	<0.0001	1,2>4
Can’t afford to have a more plant-based diet	108	16.9 (13.0–20.8)	100	13.9 (10.9–17.0)	59	9.18 (6.42–12.0)	69	8.12 (6.00–10.2)	0.0001	1>3,4; 2>4
Doesn’t like vegetarian foods	87	12.8 (9.78–15.8)	61	8.39 (5.91–10.9)	42	5.57 (3.50–7.64)	54	6.03 (4.20–7.86)	0.0004	1>3,4
The preferences of those close to one prevent from making vegetarian food	128	17.3 (13.8–20.7)	165	21.9 (18.3–25.5)	89	13.2 (10.2–16.3)	126	13.1 (10.6–15.6)	0.0003	2>3,4
Not interested in the topic	87	13.0 (9.92–16.1)	75	10.3 (7.62–13.0)	83	10.2 (7.71–12.7)	92	9.18 (7.06–11.3)	NS	NS
Some other reason	103	15.6 (12.2–18.9)	107	16.1 (12.7–19.6)	108	13.1 (10.3 15.9)	137	14.6 (12.0–17.3)	NS	NS

	Men									
	Age								General test P-value	Pair-wise comparison sign. diff.^†^
	20–39 (1)		40–54 (2)		55–64 (3)		65–74 (4)			
	n	% (95% CI)	n	% (95% CI)	n	% (95% CI)	n	% (95% CI)		
Changing diet to more plant-based	432		493		629		770			
Already done that	114	30.0 (24.5–35.5)	146	32.4 (26.9–38.0)	187	28.7 (24.3–33.1)	244	30.5 (26.6–34.3)	NS	NS
Plans to do that	63	12.1 (8.86–15.3)	84	15.4 (11.5–19.3)	99	16.1 (12.6–19.6)	179	24.2 (20.5–27.8)	<0.0001	1,2,3<4
Don’t yet plan to do that	176	38.9 (33.3–44.6)	209	41.0 (35.4–46.6)	291	47.1 (42.4–51.9)	299	39.2 (35.0–43.4)	NS	NS
Not applicable	79	19.0 (14.5–23.5)	54	11.2 (7.38–15.0)	52	8.11 (5.60–10.6)	48	6.20 (4.25–8.15)	<0.0001	1>3,4
Unsure about what food is environmentally friendly	97/434	22.5 (17.8–27.2)	108/494	24.4 (18.8–29.9)	131/627	21.9 (18.0–25.7)	194/758	25.3 (21.5–29.1)	NS	NS
Perceived social pressure to increase vegetarian food consumption	79/435	16.4 (12.3–20.5)	109/493	20.1 (15.9–24.4)	121/626	18.5 (14.8–22.1)	213/758	28.3 (24.5–32.1)	0.0001	1,2,3<4
Barriers that prevent one from making one’s diet more plant-based^‡^	437		495		634		779			
Unsure about whether a plant-based diet is nutritious enough	106	23.3 (18.5–28.0)	104	22.1 (17.2–27.1)	137	21.2 (17.3–25.0)	232	30.0 (26.1–33.9)	0.009	3<4
Doesn’t know how to prepare tasty vegetarian food	170	35.6 (30.3–40.9)	128	23.4 (18.9–27.8)	157	25.1 (21.0–29.2)	172	23.6 (20.0–27.2)	0.001	1>2,3,4
Can’t afford to have a more plant-based diet	66	16.9 (12.5–21.4)	48	9.43 (6.36–12.5)	61	10.3 (7.38–13.1)	50	7.84 (5.46–10.2)	0.005	1>2,4
Doesn’t like vegetarian foods	129	29.0 (23.6–34.3)	108	22.2 (17.4–27.0)	136	21.6 (17.8–25.5)	133	16.2 (13.2–19.2)	0.0004	1>4
The preferences of those close to one prevent from making vegetarian food	17	3.28 (1.52–5.04)	25	4.75 (2.73–6.77)	12	2.09 (0.65–3.52)	11	1.83 (0.62–3.04)	NS	NS
Not interested in the topic	152	32.5 (27.1–37.8)	146	29.5 (24.1–34.9)	156	24.7 (20.7–28.7)	181	22.1 (18.7–25.6)	0.006	1>4
Some other reason	62	14.1 (10.2–18.0)	76	11.8 (8.72–14.8)	86	13.6 (10.3–16.8)	80	9.51 (7.14–11.9)	NS	NS

NS, not statistically significant; sign. diff., significant difference.

* Population-weighted prevalences and 95% CIs.

† Considered significantly different with group rankings as indicated, if for the general test *P* < 0.05 and for pair-wise comparison *P* < 0.05.

‡ Due to small *n* and no statistically significant differences between the categories, the results concerning barriers ‘Plant-based food products are not easily available’ and ‘Doesn’t have time to change eating habits’ not presented.

## Results

### Characteristics of the study population

In all, 65% of the study population lived in urban areas, 40% lived in households with adults only, and 42% were categorised as having basic education (Table 1). In the following chapters, only statistically significant (general test *P* < 0.05, and pair-wise test *P* < 0.05, and 95% CIs not overlapping) differences between the groups are reported.

### Prevalences and differences between women and men

Nearly half of the women and one-third of the men reported to have already changed their diet to more plant-based (Table 1). Women were also more often planning to change their diet, whereas greater proportion of men reported that they do not plan to do that (men 41% vs. women 29%) or that changing diet is not applicable to them (men 13% vs. women 5%). One fifth of women and men felt uncertainty about what food is environmentally friendly. Men (20%) reported more often than women (11%) that they perceived social pressure to increase vegetarian food consumption.

**Figure 1.**
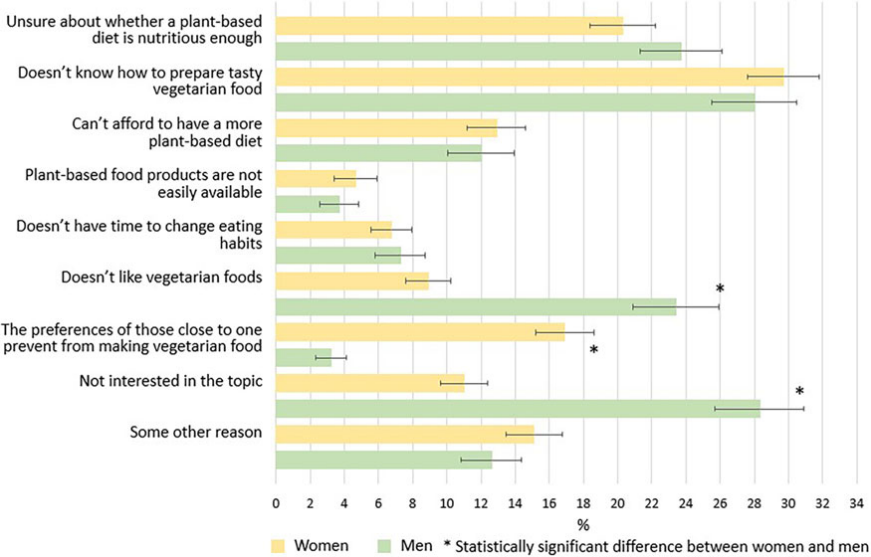
Perceived barriers to adopting more plant-based diets in women and in men.

Of women 78% and of men 86% reported at least one perceived barrier in changing their diet to more plant-based (Supplementary table 1). Roughly half of women and men reported one barrier and almost one-fifth reported two barriers, whereas reporting three or more barriers was less common. The most common barriers both in women and in men were ‘Do not know how to prepare tasty vegetarian food‘ and ’Unsure about whether a plant-based diet is nutritious enough’ (Table 1, Figure 1). In men, also ‘Do not like vegetarian foods’ and ‘Not interested in the topic’ were frequent barriers. While 17% of women reported barrier ‘The preferences of people close to me prevent me from making vegetarian food’, only 3% of men perceived this as a barrier. Barriers related to limited availability of plant-based products or lack of time to change eating habits were the least commonly perceived barriers.

### Sociodemographic group differences

**Figure 2.**
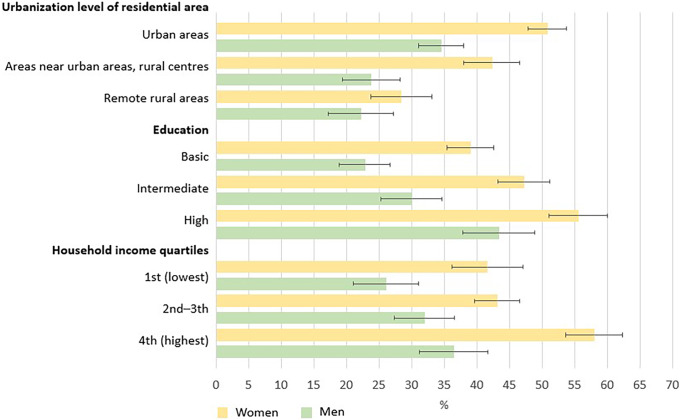
Proportions of women and men who have already changed their diet to more plant-based in different sociodemographic groups (%).

Within all studied sociodemographic groups, women had changed their diet to more plant-based more often than men had (Table 2, Supplementary tables 2–5, Figure 2). Such changes were most frequent in women and men living in urban areas (women 51%, men 35%), having high education (women 56%, men 43%), or belonging to the highest household income quartile (women 58%, men 36%).

Regarding intentions to change diet, men in the oldest age group (24%) reported planning to change their diet more often than men in the youngest age group (12%) (Table 2). In both women and men, those with basic education reported more often than those with high education that they do not plan to change their diet to more plant-based (women 34% vs. 24%; men 46% vs. 35%) (Supplementary table 4). In addition, women living in remote rural areas and women in the lowest household income quartile reported that more often than women in other corresponding groups (Supplementary tables 2 and 5).

Uncertainty about what food is environmentally friendly was more common in women (27%) and men (31%) in the lowest household income quartile, as well as in women in the youngest age group (27%), and men living in urban areas (26%), compared to other corresponding groups (Table 2, Supplementary tables 2 and 5). Men in the oldest age group (28%) and women with basic education (13%) reported more often than those in other groups that they perceived social pressure to increase vegetarian food consumption (Table 2, Supplementary table 4).

Several differences appeared between age groups in the barriers that prevent making diet more plant-based (Table 2). In both women and men, uncertainty about whether a plant-based diet is nutritious enough was most frequent in the oldest age group (women 28%, men 30 %). By contrast, in the youngest age group, both women and men reported most frequently the barrier ‘Do not know how to prepare tasty vegetarian food’ (women 34 %, men 36%). People in the youngest age group also reported more frequently than those in older age groups that they cannot afford to have a more plant-based diet or that they do not like vegetarian foods. While women aged 40–54 years reported most frequently (22%) that the preferences of people close to them prevent them from making vegetarian food, this was not an issue amongst the men with same age (5%). Barrier of disinterest in the topic was most common in men in the youngest age group (33%).

Urbanisation level of residential area showed statistically significant differences in the barrier of disinterest in the topic (Supplementary table 2). Men living in remote rural areas (34%) and women living in areas near urban areas or in rural centres (16%) reported more frequently disinterest in the topic compared to the other urbanisation level groups. A few differences in perceived barriers occurred between household structure groups (Supplementary table 3). Lack of knowledge of how to prepare tasty vegetarian food was most common in women living with children in the household (35%). Women living alone reported most frequently that they cannot afford to have a more plant-based diet. Even though the perceived barrier of ‘The preferences of those close to one prevent from making vegetarian food’ was notably more frequent in women than in men, those living in households with at least one under-age child reported this more frequently than those in other kind of households both in women (29%) and in men (6%). Education showed differences in three perceived barriers (Supplementary table 4). Women with basic education reported more often that they cannot afford to have a more plant-based diet (18%), or that they do not like vegetarian foods (11%) than women with higher education. Men with basic education (31%) reported more often than men with higher education that they are not interested in the topic. Differences by income appeared only in the perceived barrier ‘Cannot afford to have a more plant-based diet’; over one-fifth of women and men in the lowest income quartile reported this barrier (Supplementary table 5).

## Discussion

This large population-based study of Finnish adults provided up-to-date information on the proportions of individuals in different sociodemographic groups, who had changed their diet to more plant-based, or perceived different barriers to making such changes. While the basic findings were mostly compatible with the existing literature, this study also provided new detailed insights into the adoption of plant-based diets and into the specific barriers faced in several different sociodemographic groups, which have not been studied earlier in this extent.

### Adopting more plant-based diets

In this study, two-thirds of women and almost half of men had already changed or were planning to change their diet to more plant-based to combat climate change. According to a recent European consumer survey, half of the participants reported to have reduced their meat intake.^(12)^ In line with this, also some other studies have shown an increase in vegetarianism or a decrease in meat consumption during the last decades.^(33–35)^

Women had changed or were planning to change their diet to more plant-based more frequently than men had. Also earlier studies have concluded that women eat less meat and more vegetables, fruit, and other plant-based foods than men^(24,33,36)^ and have less barriers to following more plant-based diets or reducing meat consumption.^(14,20)^ Although some studies show women face more barriers in certain contexts (e.g. pulse consumption),^(16)^ they are generally more likely to adopt plant-based diets. Eating meat has traditionally been associated with masculinity, power, and wealth,^(37–39)^ which may be one explanation behind sex differences found in most of the studies. In agreement with our findings between higher socio-economic groups and more frequent adoption of more plant-based diets, a direct association between indicators of socio-economic status and plant-based food consumption,^(24,33,40)^ and an inverse association between socio-economic status and meat consumption^(23,24,36,41,42)^ have been shown also in previous studies. Other studies have also linked less sustainable attitudes or behaviour to younger age, lower education, lower income, or rural residence.^(25,31,43,44)^ This has been suggested to stem from, for instance, different values, attitudes or traditions, food literacy, lack of information on healthy lifestyle habits, or not perceiving that one would benefit from healthy lifestyle choices.^(45,46)^ In this study, differences between urban–rural axis categories may be accounted for, for example, by differences in education, traditions, attitudes, or social norms.

### Barriers to adopting more plant-based diets

In this study, most women and men reported at least one barrier to change their diet to more plant-based. Generally, barriers to following a plant-based diet have been shown to be common in men, younger age groups, rural residents, those living in households with children, and those with low education.^(14)^ Furthermore, different kinds of barriers may prevent people from changing their diets. In this study, most common barriers for women and for men were not knowing how to prepare tasty vegetarian food, and doubts about the nutritional value of a plant-based diet.

In agreement with previous findings,^(13)^ over one-fifth of the participants in this study felt uncertainty about what food is environmentally friendly or felt that uncertainty about the nutritional value of a plant-based diet prevented them from adopting it. Despite growing awareness of the health benefits of decreasing meat consumption and increasing the consumption of plant-based foods over the last decades, many studies have shown that decreasing meat consumption has not been widely seen as an important climate action.^(20,39,47,48)^ It seems that animal or plant origin of foods is not perceived as an important environmental factor, and the concept of healthy and environmentally friendly food remains unclear for many. In this study, younger women were more uncertain about environmental friendliness of food, while older participants had more doubts about the nutritional value of plant-based food. Feeling uncertainty is subjective and does not necessarily depend on the actual level of knowledge. Uncertainty about environmentally friendly food was also common in participants with the lowest income, possibly due to socio-economic differences in health literacy.^(46)^

In addition to lack of knowledge of environmentally friendly foods, also the lack of knowledge on how to prepare tasty vegetarian food, not liking vegetarian foods and the preferences of the close ones were three commonly identified barriers to adopting plant-based diets. In accordance, 30% of the participants of a recent European survey reported that taste was a barrier when choosing plant-based alternatives.^(12)^ In the present study, not knowing how to prepare tasty vegetarian food was frequently perceived as a barrier in both women and men. The lack of practical knowledge and skills to prepare such food may hinder dietary changes, but also adaptation to unfamiliar flavours and textures may require gradual exposure and time. Not knowing how to prepare tasty vegetarian food was frequent especially in younger women and in women living in households with children. Women with children may perceive that the vegetarian food they make may not meet the preferences of their children or spouses. Indeed, women – and especially women with children – perceived notably more often than men that the preferences of their close ones are a barrier to change their diet to more plant-based, while men reported notably more often than women that not liking vegetarian foods is a barrier for them. It appears that the barriers among women partly derive from preferences of their children and spouses instead of their own preferences.

In this study, affordability was not commonly perceived as a barrier to have a more plant-based diet. In previous studies, however, price and affordability factors have emerged as important drivers behind food selection.^(12,19,25,49)^ Despite the affordability not being an important barrier for all population groups in this study, this barrier was more frequent in younger adults, in women living alone, in women with low education, and in women and men with the lowest income compared to the other groups. In agreement, previous studies have emphasised the relevance of socio-economic status in the association between price and affordability and dietary choices.^(49–51)^ Especially for those with low income, price may pose a serious barrier to choose more sustainable foods.^(51)^ However, a recent optimisation study concluded that it is possible to compose a nutritionally adequate and culturally acceptable more plant-based diet while reducing the price of diet,^(52)^ which implies that with adequate know-how, barriers related to affordability could be diminished.

While approximately 10% of women reported that disinterest in the topic was a barrier to adopt a more plant-based diet, the corresponding share in men was over one quarter. Among men, approximately one-third in the youngest age group, living in remote rural areas, or with the lowest education reported lack of interest. Generally, lack of interest represents a slightly different aspect than other more pragmatic or taste-related barriers. A Dutch study concluded that consumers could be divided into four groups according to their thoughts and actions concerning meat consumption: struggling consumers, coping consumers, indifferent consumers, and strategically ignorant consumers.^(53)^ In the present study, those not interested in adopting a more plant-based diet may comprise both indifferent and strategically ignorant consumers. Either way, this group may need different kind of attention in dietary transition than those who perceive more pragmatic or taste-related barriers. Indeed, a Swedish study that explored motivations for active avoidance of carbon emission information stated that those, who are indifferent to the information offered, are not affected by it.^(54)^ Thus, providing information on sustainable dietary choices is presumably not effective among those feeling indifference, but other motivators, such as lower prices, convenience or trendiness, could help guide their food choices.

### Methodological considerations

The strengths of this study were a nationally representative study sample with a large set of questions related to barriers to adopting plant-based diets and versatile information on sociodemographic factors. This study also includes certain limitations. Firstly, despite a large population survey sample, non-participation bias may have affected the representativeness of the sub-study population. This possible bias was strived to be mitigated by using inverse probability weights that are commonly used in population surveys.^(28,32,55)^ Secondly, the term ‘plant-based diets’ was not specified in the survey questionnaire, and the participants may have understood it in various ways. However, as this study examined changes towards more plant-based diets and not any specific plant-based diet, the usage of the wide definition may be less problematic here. Thirdly, the list of barriers to adopting a more plant-based diet was pre-defined in this study and, thus, other potential barriers could not be explored. In future, qualitative studies could investigate the barriers of different groups in more depth. Fourthly, this study used categorised sociodemographic exposure variables in each case instead of continuous variables even though previous literature has indicated that categorisation into, for example, tertiles or quartiles may cause loss of power and inaccurate estimation in epidemiological studies; cause false positive findings as it leads to multiple comparisons; or hinder the comparison between studies as the cut-off points are different.^(56)^ However, in this extensive descriptive study, which aimed to identify sociodemographic groups in which dietary changes and perceived barriers to these changes are more common, categorisation was opted for as an appropriate method to enable similar comparisons of prevalences across all variables. Despite the critique, categorisation of independent variables remains to be a common procedure to treat exposure variables in epidemiological studies. In this study, the possibility of false positive findings was taken into account by using the SMM adjustment for multiple comparisons with row-wise denominator degrees of freedom in pair-wise tests. As our next step in future studies, we aim at examining these associations using continuous exposure variables. Fifthly, social desirability bias may have affected how some of the participants responded to the self-reported questions concerning sustainable dietary changes – possibly leading to slight overestimations of such socially more acceptable behaviours.^(57)^ Sixthly, this study was conducted in a cross-sectional setting, which prevented the examination of changes over time. Seventhly, this study was conducted in a Finnish context, and the results may be applicable to some other Nordic or Western countries, yet country or culture specific differences may exist.

## Conclusions

This study showed that the readiness to adopt more plant-based diets varies between sociodemographic groups, each of them facing different perceived barriers. Even though two-thirds of women and almost half of men either had changed or were planning to change their diet to more plant-based to combat climate change, a significant share, especially in certain sociodemographic groups, had no such plans. Common perceived barriers included lack of knowledge about nutritional adequacy of plant-based diets, limited cooking skills to prepare tasty vegetarian food, own or close ones’ taste preferences, affordability, and lack of interest.

Previous research has mostly concentrated to study general perceived barriers to adopt more plant-based diets in different population groups, or to study different barriers in whole study populations or in only a few population groups. This study, however, simultaneously examined the prevalence of several different barriers in several different population groups providing a more comprehensive understanding on the complex issue. The novel group-specific information, provided by this study, can be utilised to support different groups in overcoming the barriers and to tailor campaigns, nudging or other measures accordingly to increase their effectiveness within different groups. Clear and accessible information on preparing nutritionally adequate, tasty, and culturally acceptable plant-based food is central to facilitating dietary changes. To encourage this shift, it is crucial to address uncertainties about sustainable diets and make the shift easy and enjoyable. In addition to information, actions like nudging (e.g. modifying default menu options in cafeterias or placing more sustainable foods in more accessible positions in supermarkets) and fiscal policies are needed to improve the accessibility and affordability of sustainable foods. Differences between population groups in adoption and barriers may reflect justice issues, as dietary transition may not be equally accessible to all, or it may disadvantage certain groups.

Addressing the barriers requires tailored public actions, as a one-size-fits-all approach may not be effective. For instance, men who are not interested in adopting more plant-based diets, or women who perceive that the preferences of their close ones prevent them from preparing plant-based foods may require different strategies. Campaigns targeted specifically to men with the most resistance could underline taste and protein content, while campaigns to whole families could underline taste, convenience and joy of eating together.

Small, gradual, and manageable changes in diet along with clear information on sustainable diets and personal benefits related to them, as well as enhanced availability of plant-based foods, are essential to dietary transition. Food industry, retail, catering services, media, and education have a significant role in promoting healthy and sustainable diets. Addressing the characteristics and perceived barriers of the groups, whose diets are far from meeting dietary guidelines, is vital for reducing sociodemographic differences in diets. Ensuring that dietary transition is accessible to all sociodemographic groups is crucial for planetary health and future well-being.

## Supporting information

10.1017/jns.2026.10101.sm001 Sares-Jäske et al. supplementary material Sares-Jäske et al. supplementary material

## Data Availability

The data that support the findings of this study are available from the Finnish Social and Health Data Permit Authority Findata. Restrictions apply to the availability of these data, which were used under licence for this study. Data are available at https://findata.fi/en/ with the permission of Findata.

## Supplementary material

The supplementary material for this article can be found at https://doi.org/10.1017/jns.2026.10101.
