# Supplementary material for: Perceived barriers to adopting more plant-based diets across sociodemographic groups: findings from a population-based survey in Finland
Source: J Nutr Sci. 2026 May 20;15:e35. doi: 10.1017/jns.2026.10101 (PMC13200019; doi:10.1017/jns.2026.10101)
Supplement: Sares-Jäske et al. supplementary material [file S2048679026101013sup001.docx]

| **Supplementary table 1.** Distributions (population-weighted prevalences and 95% confidence intervals (CI)) of study population in categories of variables of interest (Healthy Finland Survey, n=5390). | | | | | | |
| --- | --- | --- | --- | --- | --- | --- |
|  | **Women** | |  | **Men** | |  |
| *Attitudes towards plant-based diet* | **n** | **% (95% CI)** | | **n** | **% (95% CI)** | |
| Changing diet to more plant-based | 3028 |  | | 2324 |  | |
| Already done that | 1385 | 46.3 (44.0-48.6) | | 691 | 30.5 (27.8-33.2) | |
| Plans to do that | 645 | 20.2 (18.4-22.1) | | 425 | 15.9 (14.0-17.7) | |
| Don’t yet plan to do that | 877 | 28.7 (26.6-30.8) | | 975 | 41.1 (38.3-43.9) | |
| Not applicable | 121 | 4.73 (3.62-5.84) | | 233 | 12.5 (10.5-14.5) | |
|  |  |  | |  |  | |
| Unsure about what food is environmentally friendly | 3004 |  | | 2313 |  | |
| Strongly disagree | 856 | 28.0 (25.9-30.0) | | 589 | 26.6 (24.1-29.1) | |
| Somewhat disagree | 957 | 32.0 (29.8-34.1) | | 634 | 27.9 (25.4-30.4) | |
| Neither agree nor disagree | 574 | 18.2 (16.4-19.9) | | 560 | 22.2 (19.8-24.6) | |
| Somewhat agree | 549 | 19.4 (17.5-21.3) | | 419 | 17.8 (15.7-20.0) | |
| Strongly agree | 68 | 2.52 (1.68-3.37) | | 111 | 5.54 (3.97-7.11) | |
|  |  |  | |  |  | |
| Perceived social pressure to increase vegetarian food consumption | 3001 |  | | 2312 |  | |
| Strongly disagree | 1466 | 49.0 (46.7-51.3) | | 706 | 34.3 (31.5-37.1) | |
| Somewhat disagree | 501 | 16.7 (15.0-18.5) | | 416 | 18.0 (15.7-20.3) | |
| Neither agree nor disagree | 712 | 23.3 (21.3-25.3) | | 668 | 27.8 (25.3-30.2) | |
| Somewhat agree | 241 | 7.84 (6.53-9.15) | | 424 | 16.0 (14.0-18.0) | |
| Strongly agree | 81 | 3.15 (2.20-4.10) | | 98 | 3.90 (2.94-4.85) | |
|  |  |  | |  |  | |
| Barriers that prevent one from making one’s diet more plant-based, % | 3045 |  | | 2345 |  | |
| Unsure about whether a plant-based diet is nutritious enough | 660 | 20.3 (18.4-22.1) | | 579 | 23.7 (21.3-26.1) | |
| Doesn’t know how to prepare tasty vegetarian food | 874 | 29.7 (27.6-31.8) | | 627 | 28.0 (25.6-30.5) | |
| Can’t afford to have a more plant-based diet | 336 | 12.9 (11.2-14.6) | | 225 | 12.0 (10.0-13.9) | |
| Plant-based food products are not easily available | 119 | 4.66 (3.41-5.92) | | 77 | 3.69 (2.56-4.81) | |
| Doesn’t have time to change eating habits | 182 | 6.78 (5.57-7.98) | | 139 | 7.28 (5.83-8.74) | |
| Doesn’t like vegetarian foods | 244 | 8.89 (7.55-10.2) | | 506 | 23.4 (20.9-25.9) | |
| The preferences of those close to one prevent from making vegetarian food | 508 | 16.9 (15.2-18.6) | | 65 | 3.21 (2.30-4.11) | |
| Not interested in the topic | 337 | 11.0 (9.56-12.4) | | 635 | 28.3 (25.7-30.9) | |
| Some other reason | 455 | 15.1 (13.4-16.7) | | 304 | 12.6 (10.8-14.3) | |
|  |  |  | |  |  | |
| At least one of the preceding barriers | 3045 | 78.2 (76.3-80.1) | | 2345 | 85.6 (83.4-87.8) | |
|  |  |  | |  |  | |
| Sum of the preceding barriers | 3045 |  | | 2345 |  | |
| 0 | 639 | 21.8 (19.9-23.7) | | 325 | 14.4 (12.2-16.6) | |
| 1 | 1553 | 48.1 (45.8-50.4) | | 1301 | 51.0 (48.2-53.9) | |
| 2 | 528 | 17.3 (15.6-19.0) | | 411 | 19.2 (16.9-21.5) | |
| 3 | 229 | 9.20 (7.77-10.6) | | 224 | 10.8 (9.08-12.6) | |
| 4 | 69 | 2.55 (1.65-3.44) | | 61 | 2.76 (1.85-3.68) | |
| 5 | 21 | 0.88 (0.45-1.32) | | 21 | 1.56 (0.75-2.38) | |
| 6 | 5 | 0.20 (0.00-0.42) | | 1 | 0.15 (0.00-0.45) | |
| 7 | 0 | - | | 1 | 0.07 (0.00-0.22) | |
| 8 | 1 | 0.02 (0.00-0.05) | | 0 | - | |
|  |  |  | |  |  | |
| Sum of the preceding barriers, mean (95% CI) | 3045 | 1.26 (1.21-1.31) | | 2345 | 1.42 (1.36-1.49) | |
| Abbreviations: CI, Confidence interval. | | | | | | |

| **Supplementary table 2.** Prevalences and 95% confidence intervals of studied factors related to adopting a more plant-based diet in urbanization level of residential area categories in women and in men (n=5390)^*^. | | | | | | | | | | | | | | | | | | | | | | | | |
| --- | --- | --- | --- | --- | --- | --- | --- | --- | --- | --- | --- | --- | --- | --- | --- | --- | --- | --- | --- | --- | --- | --- | --- | --- |
|  | **Women** | | | | | | | | | | |  | **Men** | | | | | | | | | | |  |
|  | **Urbanization level of residential area** | | | | | | | |  | **General test**  **P-value** | **Pair-wise comparison**  **sign. diff.^†^** | | **Urbanization level of residential area** | | | | | | | |  | **General test**  **P-value** | **Pair-wise comparison**  **sign. diff.^†^** | |
|  | **Urban areas (1)** | |  | **Areas near urban areas, rural centres (2)** | |  | **Remote rural areas (3)** | |  |  |  |  | **Urban areas (1)** | |  | **Areas near urban areas, rural centres (2)** | |  | **Remote rural areas (3)** | |  |  |  |  |
|  | **n** | **% (95% CI)** | | **n** | **% (95% CI)** | | **n** | **% (95% CI)** | |  |  |  | **n** | **% (95% CI)** | | **n** | **% (95% CI)** | | **n** | **% (95% CI)** | |  |  |  |
| Changing diet to more plant-based | 1725 |  | | 777 |  | | 526 |  | |  |  | | 1282 |  | | 596 |  | | 446 |  | |  |  | |
| Already done that | 858 | 50.8 (47.8-53.7) | | 352 | 42.3 (38.0-46.6) | | 175 | 28.4 (23.7-33.1) | | <0.0001 | 1>2,3; 2>3 | | 422 | 34.5 (31.0-38.0) | | 164 | 23.8 (19.3-28.2) | | 105 | 22.2 (17.2-27.2) | | <0.0001 | 1>2,3 | |
| Plans to do that | 364 | 20.6 (18.2-23.0) | | 164 | 19.3 (16.1-22.5) | | 117 | 22.5 (17.8-27.2) | | NS | NS | | 236 | 16.1 (13.7-18.5) | | 115 | 18.1 (14.4-21.8) | | 74 | 15.8 (11.4-20.1) | | NS | NS | |
| Don’t yet plan to do that | 448 | 24.8 (22.2-27.4) | | 227 | 33.1 (28.9-37.3) | | 202 | 40.3 (34.9-45.7) | | <0.0001 | 1<2,3 | | 517 | 40.0 (36.4-43.7) | | 249 | 41.7 (36.6-46.8) | | 209 | 43.2 (37.3-49.1) | | NS | NS | |
| Not applicable | 55 | 3.93 (2.60-5.26) | | 34 | 5.30 (3.26-7.33) | | 32 | 8.85 (5.37-12.3) | | 0.028 | 1<3 | | 107 | 9.37 (7.00-11.7) | | 68 | 16.4 (12.1-20.8) | | 58 | 18.9 (13.7-24.0) | | 0.0007 | 1<2,3 | |
| Unsure about what food is environmentally friendly | 383/  1722 | 22.6 (20.1-25.2) | | 123/  764 | 18.1 (14.7-21.5) | | 111/  518 | 24.3 (19.1-29.6) | | 0.049 | NS | | 314/  1280 | 26.1 (22.8-29.4) | | 130/  592 | 21.0 (17.0-25.0) | | 86/  441 | 18.2 (13.9-22.5) | | 0.009 | 1>3 | |
| Perceived social pressure to increase vegetarian food consumption | 173/  1724 | 11.5 (9.42-13.5) | | 78/  762 | 8.83 (6.48-11.2) | | 71/  515 | 12.3 (9.06-15.5) | | NS | NS | | 273/  1282 | 20.3 (17.6-23.1) | | 130/  591 | 19.7 (15.9-23.5) | | 119/  439 | 25.1 (19.8-30.3) | | NS | NS | |
| Barriers that prevent one from making one’s diet more plant-based^‡^ | 1739 |  | | 779 |  | | 527 |  | |  |  | | 1295 |  | | 599 |  | | 451 |  | |  |  | |
| Unsure about whether a plant-based diet is nutritious enough | 394 | 21.7 (19.3-24.1) | | 152 | 19.3 (16.0-22.5) | | 114 | 19.2 (15.3-23.0) | | NS | NS | | 332 | 25.8 (22.7-28.9) | | 141 | 21.8 (17.9-25.7) | | 106 | 22.4 (17.4-27.4) | | NS | NS | |
| Doesn’t know how to prepare tasty vegetarian food | 540 | 30.5 (27.8-33.2) | | 216 | 29.1 (25.2-33.0) | | 118 | 23.6 (19.0-28.2) | | 0.037 | 1>3 | | 370 | 28.7 (25.5-31.8) | | 163 | 29.0 (24.3-33.6) | | 94 | 23.3 (18.1-28.4) | | NS | NS | |
| Can’t afford to have a more plant-based diet | 182 | 11.9 (9.78-14.1) | | 81 | 11.9 (9.02-14.7) | | 73 | 18.2 (13.8-22.5) | | 0.030 | 1,2<3 | | 127 | 12.0 (9.63-14.5) | | 55 | 11.1 (8.02-14.2) | | 43 | 12.6 (8.43-16.8) | | NS | NS | |
| Doesn’t like vegetarian foods | 146 | 8.48 (6.83-10.1) | | 56 | 8.82 (6.23-11.4) | | 42 | 9.79 (6.60-13.0) | | NS | NS | | 270 | 23.0 (19.8-26.2) | | 140 | 24.8 (20.4-29.2) | | 96 | 21.6 (16.6-26.6) | | NS | NS | |
| The preferences of those close to one prevent from making vegetarian food | 288 | 16.2 (14.0-18.4) | | 134 | 16.9 (13.7-20.1) | | 86 | 18.5 (14.0-23.0) | | NS | NS | | 42 | 3.38 (2.18-4.58) | | 18 | 3.49 (1.67-5.30) | | 5 | 1.59 (0.47-3.13) | | NS | NS | |
| Not interested in the topic | 163 | 8.88 (7.19-10.6) | | 100 | 16.1 (12.6-19.6) | | 74 | 13.8 (10.4-17.2) | | 0.0003 | 1<2,3 | | 320 | 25.0 (21.7-28.3) | | 178 | 32.7 (27.8-37.7) | | 137 | 34.2 (28.5-39.9) | | 0.005 | 1<2,3 | |
| Some other reason | 245 | 13.5 (11.5-15.5) | | 118 | 16.8 (13.2-20.4) | | 92 | 21.6 (16.8-26.5) | | 0.007 | 1<3 | | 167 | 11.2 (9.03-13.4) | | 68 | 11.6 (8.17-14.9) | | 69 | 16.0 (11.5-20.4) | | NS | NS | |
| Abbreviations: CI, Confidence interval; NS, not statistically significant; sign. diff., significant difference. | | | | | | | | | | | | | | | | | | | | | | | | |
| ^*^ Age-adjusted and population-weighted prevalences and 95% confidence intervals. | | | | | | | | | | | | | | | | | | | | | | | | |
| ^†^ Considered significantly different with group rankings as indicated, if for the general test p<0.05 and for pair-wise comparison p<0.05. | | | | | | | | | | | | | | | | | | | | | | | | |
| ^‡^ Due to small n and no statistically significant differences between the categories, the results concerning barriers “Plant-based food products are not easily available” and “Doesn’t have time to change eating habits” not presented. | | | | | | | | | | | | | | | | | | | | | | | | |

| **Supplementary table 3.** Prevalences and 95% confidence intervals of studied factors related to adopting a more plant-based diet in household structure categories in women and in men (n=4681)^*^. | | | | | | | | | | | | | | | | | | | | | | | | |
| --- | --- | --- | --- | --- | --- | --- | --- | --- | --- | --- | --- | --- | --- | --- | --- | --- | --- | --- | --- | --- | --- | --- | --- | --- |
|  | **Women** | | | | | | | | | | |  | **Men** | | | | | | | | | | |  |
|  | **Household structure** | | | | | | | |  | **General test**  **P-value** | **Pair-wise comparison**  **sign. diff.^†^** | | **Household structure** | | | | | | | |  | **General test**  **P-value** | **Pair-wise comparison**  **sign. diff.^†^** | |
|  | **Living alone (1)** | |  | **Adults only (2)** | |  | **At least one adult and one child (3)** | |  |  |  |  | **Living alone (1)** | |  | **Adults only (2)** | |  | **At least one adult and one child (3)** | |  |  |  |  |
|  | **n** | **% (95% CI)** | | **n** | **% (95% CI)** | | **n** | **% (95% CI)** | |  |  |  | **n** | **% (95% CI)** | | **n** | **% (95% CI)** | | **n** | **% (95% CI)** | |  |  |  |
| Changing diet to more plant-based^2^ | 776 |  | | 1232 |  | | 629 |  | |  |  | | 560 |  | | 998 |  | | 455 |  | |  |  | |
| Already done that | 380 | 49.8 (45.0-54.6) | | 587 | 48.9 (44.7-52.5) | | 250 | 40.9 (35.1-46.7) | | NS | NS | | 141 | 26.1 (21.3-31.0) | | 323 | 33.4 (28.6-38.2) | | 140 | 34.0 (27.5-40.5) | | NS | NS | |
| Plans to do that | 153 | 18.3 (14.5-22.1) | | 253 | 18.6 (15.8-21.5) | | 147 | 23.7 (19.3-28.2) | | NS | NS | | 101 | 16.2 (12.5-19.8) | | 166 | 13.1 (10.5-15.6) | | 92 | 19.2 (14.9-23.6) | | NS | NS | |
| Don’t yet plan to do that | 202 | 26.5 (22.0-30.9) | | 357 | 29.6 (25.8-33.3) | | 208 | 30.2 (24.8-35.5) | | NS | NS | | 260 | 44.9 (39.4-50.4) | | 415 | 40.1 (35.4-44.8) | | 171 | 36.9 (30.6-43.1) | | NS | NS | |
| Not applicable | 41 | 5.41 (3.50-7.32) | | 35 | 3.24 (1.77-4.70) | | 24 | 5.25 (2.92-7.58) | | NS | NS | | 58 | 12.8 (8.81-16.8) | | 94 | 13.4 (9.70-17.2) | | 52 | 9.97 (5.71-14.2) | | NS | NS | |
| Unsure about what food is environmentally friendly | 163/  762 | 21.9 (17.8-25.9) | | 222/  1219 | 19.1 (16.0-22.2) | | 149/  632 | 25.3 (20.3-30.2) | | NS | NS | | 148/  560 | 28.4 (23.4-33.3) | | 217/  997 | 21.9 (17.6-26.1) | | 93/  455 | 21.9 (16.1-27.7) | | NS | NS | |
| Perceived social pressure to increase vegetarian food consumption | 95/  762 | 13.4 (9.88-16.9) | | 128/  1218 | 9.56 (7.31-11.8) | | 52/  631 | 7.95 (4.64-11.3) | | NS | NS | | 113/  556 | 17.2 (13.5-20.9) | | 234/  995 | 21.6 (17.8-25.5) | | 108/  456 | 25.8 (20.8-30.8) | | 0.020 | 1<3 | |
| Barriers that prevent one from making one’s diet more plant-based^‡^ | 783 |  | | 1235 |  | | 632 |  | |  |  | | 567 |  | | 1006 |  | | 458 |  | |  |  | |
| Unsure about whether a plant-based diet is nutritious enough | 194 | 22.3 (18.5-26.0) | | 275 | 20.5 (17.5-23.4) | | 103 | 19.9 (15.5-24.3) | | NS | NS | | 143 | 23.4 (19.0-27.8) | | 275 | 28.9 (24.3-33.5) | | 89 | 20.6 (15.4-25.8) | | NS | NS | |
| Doesn’t know how to prepare tasty vegetarian food | 196 | 26.7 (22.4-31.0) | | 319 | 26.3 (22.9-29.7) | | 241 | 35.2 (29.8-40.6) | | 0.036 | 2<3 | | 190 | 32.0 (27.2-36.9) | | 231 | 26.1 (21.8-30.4) | | 129 | 26.2 (20.7-31.8) | | NS | NS | |
| Can’t afford to have a more plant-based diet | 118 | 19.1 (14.6-23.5) | | 99 | 9.74 (7.28-12.2) | | 84 | 9.81 (5.84-13.8) | | 0.0005 | 1>2,3 | | 83 | 15.5 (11.6-19.4) | | 71 | 8.84 (5.94-11.7) | | 51 | 13.2 (8.89-17.5) | | 0.022 | 1>2 | |
| Doesn’t like vegetarian foods | 79 | 10.0 (7.43-12.6) | | 81 | 8.22 (5.75-10.7) | | 60 | 8.37 (4.95-11.8) | | NS | NS | | 134 | 24.4 (19.7-29.0) | | 203 | 22.5 (18.4-26.7) | | 100 | 21.5 (16.2-26.9) | | NS | NS | |
| The preferences of those close to one prevent from making vegetarian food | 27 | 4.05 (1.97-6.13) | | 223 | 18.4 (15.5-21.2) | | 198 | 28.6 (24.1-33.2) | | <0.0001 | 1<2,3; 2<3 | | 6 | 1.44 (0.14-2.74) | | 25 | 2.82 (1.49-4.15) | | 29 | 6.43 (3.86-9.01) | | 0.002 | 1,2<3 | |
| Not interested in the topic | 83 | 9.65 (7.16-12.1) | | 130 | 10.8 (7.97-13.5) | | 81 | 13.3 (9.65-16.9) | | NS | NS | | 160 | 28.6 (23.6-33.6) | | 258 | 27.6 (23.0-32.2) | | 139 | 26.6 (20.9-32.3) | | NS | NS | |
| Some other reason | 132 | 16.8 (13.4-20.2) | | 181 | 15.5 (12.5-18.5) | | 85 | 14.5 (9.84-19.1) | | NS | NS | | 53 | 7.70 (4.97-10.4) | | 134 | 12.9 (9.98-15.8) | | 71 | 14.3 (10.1-18.5) | | 0.010 | 1<2,3 | |
| Abbreviations: CI, Confidence interval; NS, not statistically significant; sign. diff., significant difference. | | | | | | | | | | | | | | | | | | | | | | | | |
| ^*^ Age-adjusted and population-weighted prevalences and 95% confidence intervals. | | | | | | | | | | | | | | | | | | | | | | | | |
| ^†^ Considered significantly different with group rankings as indicated, if for the general test p<0.05 and for pair-wise comparison p<0.05. | | | | | | | | | | | | | | | | | | | | | | | | |
| ^‡^ Due to small n and no statistically significant differences between the categories, the results concerning barriers “Plant-based food products are not easily available” and “Doesn’t have time to change eating habits” not presented. | | | | | | | | | | | | | | | | | | | | | | | | |

| **Supplementary table 4.** Prevalences and 95% confidence intervals of studied factors related to adopting a more plant-based diet in educational categories in women and in men (n=5289)^*^. | | | | | | | | | | | | | | | | | | | | | | | | |
| --- | --- | --- | --- | --- | --- | --- | --- | --- | --- | --- | --- | --- | --- | --- | --- | --- | --- | --- | --- | --- | --- | --- | --- | --- |
|  | **Women** | | | | | | | | | | |  | **Men** | | | | | | | | | | |  |
|  | **Education** | | | | | | | |  | **General**  **test**  **P-value** | **Pair-wise comparison**  **sign. diff.^†^** | | **Education** | | | | | | | |  | **General**  **test**  **P-value** | **Pair-wise comparison**  **sign. diff.^†^** | |
|  | **Basic (1)** | |  | **Intermediate (2)** | |  | **High (3)** | |  |  |  |  | **Basic (1)** | |  | **Intermediate (2)** | |  | **High (3)** | |  |  |  |  |
|  | **n** | **% (95% CI)** | | **n** | **% (95% CI)** | | **n** | **% (95% CI)** | |  |  |  | **n** | **% (95% CI)** | | **n** | **% (95% CI)** | | **n** | **% (95% CI)** | |  |  |  |
| Changing diet to more plant-based^2^ | 1159 |  | | 983 |  | | 831 |  | |  |  | | 934 |  | | 694 |  | | 653 |  | |  |  | |
| Already done that | 446 | 39.0 (35.5-42.6) | | 451 | 47.2 (43.2-51.2) | | 465 | 55.5 (51.0-60.0) | | <0.0001 | 1<2,3; 2<3 | | 215 | 22.8 (18.9-26.7) | | 201 | 30.0 (25.3-34.7) | | 266 | 43.4 (37.9-48.9) | | <0.0001 | 1,2<3 | |
| Plans to do that | 246 | 20.2 (17.4-23.0) | | 232 | 22.3 (18.8-25.8) | | 156 | 19.0 (15.5-22.5) | | NS | NS | | 170 | 15.8 (13.0-18.5) | | 125 | 17.4 (14.1-20.7) | | 116 | 16.1 (12.4-19.8) | | NS | NS | |
| Don’t yet plan to do that | 407 | 33.8 (30.4-37.2) | | 258 | 25.7 (22.3-29.1) | | 195 | 23.7 (19.6-27.8) | | 0.0002 | 1>2,3 | | 439 | 46.0 (41.6-50.4) | | 289 | 39.3 (34.5-44.2) | | 232 | 34.6 (29.2-40.1) | | 0.005 | 1>3 | |
| Not applicable | 60 | 6.95 (4.76-9.14) | | 42 | 4.86 (3.12-6.60) | | 15 | 1.80 (0.64-2.95) | | <0.0001 | 1,2>3 | | 110 | 15.4 (12.0-18.9) | | 79 | 13.3 (9.66-17.0) | | 39 | 5.89 (3.46-8.32) | | <0.0001 | 1,2>3 | |
| Unsure about what food is environmentally friendly | 268/  1143 | 24.1 (20.8-27.3) | | 205/  979 | 23.0 (19.4-26.7) | | 136/  830 | 18.0 (14.4-21.5) | | 0.036 | 1>3 | | 230/  928 | 26.3 (22.2-30.5) | | 154/  695 | 25.1 (20.5-29.7) | | 133/  647 | 18.7 (14.9-22.4) | | 0.014 | 1>3 | |
| Perceived social pressure to increase vegetarian food consumption | 153/  1138 | 13.2 (10.6-15.8) | | 97/  978 | 11.4 (8.22-14.6) | | 61/  833 | 7.14 (4.96-9.31) | | 0.002 | 1>3 | | 242/  923 | 23.5 (20.0-27.0) | | 145/  696 | 20.1 (16.3-23.9) | | 125/  651 | 17.4 (13.8-21.1) | | NS | NS | |
| Barriers that prevent one from making one’s diet more plant-based^‡^ | 1166 |  | | 986 |  | | 836 |  | |  |  | | 945 |  | | 702 |  | | 654 |  | |  |  | |
| Unsure about whether a plant-based diet is nutritious enough | 250 | 19.3 (16.6-22.0) | | 229 | 23.1 (19.8-26.4) | | 168 | 20.7 (17.0-24.4) | | NS | NS | | 236 | 23.3 (19.6-26.9) | | 167 | 25.7 (21.3-30.2) | | 164 | 24.8 (20.3-29.3) | | NS | NS | |
| Doesn’t know how to prepare tasty vegetarian food | 322 | 28.6 (25.3-31.9) | | 312 | 33.4 (29.6-37.2) | | 232 | 26.4 (22.5-30.4) | | 0.035 | 2>3 | | 232 | 27.1 (23.3-31.0) | | 209 | 29.7 (25.3-34.1) | | 182 | 28.7 (23.9-33.6) | | NS | NS | |
| Can’t afford to have a more plant-based diet | 178 | 18.0 (15.0-20.9) | | 84 | 9.53 (6.90-12.2) | | 70 | 8.75 (5.76-11.7) | | <0.0001 | 1>2,3 | | 110 | 14.0 (10.8-17.1) | | 64 | 10.6 (7.29-14.0) | | 47 | 10.3 (6.84-13.7) | | NS | NS | |
| Doesn’t like vegetarian foods | 109 | 11.0 (8.60-13.3) | | 81 | 8.12 (5.96-10.3) | | 48 | 5.74 (3.55-7.93) | | 0.008 | 1>3 | | 219 | 26.4 (22.5-30.3) | | 161 | 22.7 (18.6-26.8) | | 120 | 18.9 (14.0-23.9) | | NS | NS | |
| The preferences of those close to one prevent from making vegetarian food | 169 | 15.0 (12.3-17.7) | | 188 | 19.2 (16.2-22.3) | | 150 | 17.1 (13.7-20.4) | | NS | NS | | 18 | 2.38 (1.05-3.71) | | 26 | 3.57 (1.91-5.23) | | 20 | 3.89 (2.03-5.75) | | NS | NS | |
| Not interested in the topic | 149 | 12.8 (10.4-15.3) | | 104 | 10.5 (8.09-12.8) | | 73 | 8.96 (6.30-11.6) | | NS | NS | | 281 | 31.0 (26.8-35.1) | | 188 | 29.2 (24.6-33.7) | | 147 | 20.7 (16.0-25.4) | | 0.005 | 1,2>3 | |
| Some other reason | 183 | 16.2 (13.5-18.9) | | 132 | 13.8 (10.9-16.6) | | 137 | 16.0 (12.7-19.2) | | NS | NS | | 97 | 9.64 (7.21-12.1) | | 110 | 14.9 (11.4-18.4) | | 96 | 12.6 (9.24-16.1) | | 0.049 | NS | |
| Abbreviations: CI, Confidence interval; NS, not statistically significant; sign. diff., significant difference. | | | | | | | | | | | | | | | | | | | | | | | | |
| ^*^ Age-adjusted and population-weighted prevalences and 95% confidence intervals. | | | | | | | | | | | | | | | | | | | | | | | | |
| ^†^ Considered significantly different with group rankings as indicated, if for the general test p<0.05 and for pair-wise comparison p<0.05. | | | | | | | | | | | | | | | | | | | | | | | | |
| ^‡^ Due to small n and no statistically significant differences between the categories, the results concerning barriers “Plant-based food products are not easily available” and “Doesn’t have time to change eating habits” not presented. | | | | | | | | | | | | | | | | | | | | | | | | |

| **Supplementary table 5.** Prevalences and 95% confidence intervals of studied factors related to adopting a more plant-based diet in income categories in women and in men (n=4629)^*^. | | | | | | | | | | | | | | | | | | | | | | | | |
| --- | --- | --- | --- | --- | --- | --- | --- | --- | --- | --- | --- | --- | --- | --- | --- | --- | --- | --- | --- | --- | --- | --- | --- | --- |
|  | **Women** | | | | | | | | | | |  | **Men** | | | | | | | | | | |  |
|  | **Income quartiles** | | | | | | | |  | **General**  **test**  **P-value** | **Pair-wise comparison**  **sign. diff.^†^** | | **Income quartiles** | | | | | | | |  | **General**  **test**  **P-value** | **Pair-wise comparison**  **sign. diff.^†^** | |
|  | **1st (1)** | |  | **2nd–3th (2)** | |  | **4^th^ (3)** | |  |  |  |  | **1st (1)** | |  | **2nd–3th (2)** | |  | **4^th^ (3)** | |  |  |  |  |
|  | **n** | **% (95% CI)** | | **n** | **% (95% CI)** | | **n** | **% (95% CI)** | |  |  |  | **n** | **% (95% CI)** | | **n** | **% (95% CI)** | | **n** | **% (95% CI)** | |  |  |  |
| Changing diet to more plant-based^2^ | 516 |  | | 1265 |  | | 824 |  | |  |  | | 527 |  | | 886 |  | | 584 |  | |  |  | |
| Already done that | 211 | 41.6 (36.1-47.0) | | 545 | 43.1 (39.6-46.6) | | 449 | 58.0 (53.6-62.4) | | <0.0001 | 1,2<3 | | 138 | 26.0 (21.0-31.0) | | 261 | 32.0 (27.4-36.6) | | 204 | 36.4 (31.2-41.7) | | 0.018 | 1<3 | |
| Plans to do that | 102 | 17.7 (13.6-21.8) | | 286 | 21.5 (18.5-24.4) | | 159 | 19.2 (15.8-22.6) | | NS | NS | | 97 | 17.8 (13.9-21.7) | | 164 | 14.7 (12.0-17.5) | | 95 | 14.5 (11.0-17.9) | | NS | NS | |
| Don’t yet plan to do that | 175 | 34.4 (29.1-39.8) | | 382 | 30.3 (27.0-33.6) | | 199 | 21.3 (17.5-25.1) | | 0,0001 | 1,2>3 | | 211 | 36.5 (31.2-41.9) | | 383 | 42.6 (37.9-47.3) | | 241 | 42.3 (36.8-47.8) | | NS | NS | |
| Not applicable | 28 | 6.32 (3.64-8.99) | | 52 | 5.15 (3.43-6.87) | | 17 | 1.53 (0.37-2.68) | | 0.0003 | 1,2>3 | | 81 | 19.7 (14.6-24.7) | | 78 | 10.6 (7.65-13.6) | | 44 | 6.80 (4.05-9.54) | | 0.0001 | 1>2,3 | |
| Unsure about what food is environmentally friendly | 134/  505 | 27.1 (22.1-32.1) | | 239/  1251 | 19.1 (16.2-22.0) | | 151/  825 | 20.8 (17.2-24.3) | | 0.030 | 1>2 | | 151/  528 | 30.6 (25.3-35.9) | | 197/  883 | 21.9 (17.9-25.8) | | 106/  584 | 20.2 (15.4-25.0) | | 0.011 | 1>2,3 | |
| Perceived social pressure to increase vegetarian food consumption | 73/  506 | 12.7 (9.32-16.0) | | 131/  1249 | 10.9 (8.36-13.3) | | 65/  825 | 7.51 (5.14-9.89) | | 0.032 | 1>3 | | 124/  527 | 21.3 (17.0-25.5) | | 215/  881 | 23.2 (19.3-27.1) | | 107/  583 | 16.3 (12.5-20.2) | | 0.037 | 2>3 | |
| Barriers that prevent one from making one’s diet more plant-based^‡^ | 520 |  | | 1269 |  | | 827 |  | |  |  | | 536 |  | | 891 |  | | 586 |  | |  |  | |
| Unsure about whether a plant-based diet is nutritious enough | 105 | 19.5 (15.2-23.9) | | 303 | 22.9 (19.9-25.8) | | 160 | 19.4 (16.1-22.7) | | NS | NS | | 160 | 28.1 (23.1-33.0) | | 224 | 23.7 (19.7-27.6) | | 116 | 22.7 (17.9-27.6) | | NS | NS | |
| Doesn’t know how to prepare tasty vegetarian food | 143 | 25.1 (20.4-29.9) | | 348 | 30.2 (26.8-33.6) | | 262 | 29.9 (26.0-33.8) | | NS | NS | | 138 | 28.0 (23.0-33.0) | | 249 | 28.1 (24.1-32.2) | | 161 | 28.9 (24.1-33.8) | | NS | NS | |
| Can’t afford to have a more plant-based diet | 118 | 23.7 (18.7-28.7) | | 152 | 13.8 (11.1-16.5) | | 29 | 2.46 (0.87-4.05) | | <0.0001 | 1>2,3; 2>3 | | 106 | 22.6 (17.8-27.4) | | 77 | 9.66 (6.94-12.4) | | 21 | 4.02 (1.67-6.37) | | <0.0001 | 1>2,3; 2>3 | |
| Doesn’t like vegetarian foods | 67 | 10.7 (7.43-13.9) | | 100 | 9.64 (7.24-12.0) | | 47 | 5.74 (3.66-7.82) | | 0.010 | 1>3 | | 126 | 24.0 (19.1-28.9) | | 180 | 21.9 (17.8-25.9) | | 126 | 23.5 (18.9-28.1) | | NS | NS | |
| The preferences of those close to one prevent from making vegetarian food | 75 | 13.8 (10.0-17.6) | | 194 | 15.3 (12.9-17.8) | | 173 | 20.3 (16.8-23.9) | | 0.037 | NS | | 11 | 2.77 (0.99-4.55) | | 27 | 3.25 (1.84-4.66) | | 21 | 3.23 (1.59-4.87) | | NS | NS | |
| Not interested in the topic | 66 | 12.2 (8.78-15.6) | | 143 | 10.5 (8.41-12.5) | | 80 | 10.6 (7.53-13.7) | | NS | NS | | 149 | 26.6 (21.5-31.7) | | 229 | 27.4 (23.3-31.6) | | 173 | 29.0 (23.9-34.0) | | NS | NS | |
| Some other reason | 80 | 16.9 (12.7-21.2) | | 193 | 14.8 (12.3-17.3) | | 122 | 16.4 (13.1-19.7) | | NS | NS | | 62 | 11.6 (8.20-15.0) | | 114 | 11.3 (8.54-14.1) | | 81 | 11.3 (8.12-14.5) | | NS | NS | |
| Abbreviations: CI, Confidence interval; NS, not statistically significant; sign. diff., significant difference. | | | | | | | | | | | | | | | | | | | | | | | | |
| ^*^ Age-adjusted and population-weighted prevalences and 95% confidence intervals. | | | | | | | | | | | | | | | | | | | | | | | | |
| ^†^ Considered significantly different with group rankings as indicated, if for the general test p<0.05 and for pair-wise comparison p<0.05. | | | | | | | | | | | | | | | | | | | | | | | | |
| ^‡^ Due to small n and no statistically significant differences between the categories, the results concerning barriers “Plant-based food products are not easily available” and “Doesn’t have time to change eating habits” not presented. | | | | | | | | | | | | | | | | | | | | | | | | |
